# Association of *CYP2D6* metabolizer status with mammographic density change in response to tamoxifen treatment

**DOI:** 10.1186/bcr3495

**Published:** 2013-10-03

**Authors:** Jingmei Li, Kamila Czene, Hiltrud Brauch, Werner Schroth, Pilar Saladores, Yi Li, Keith Humphreys, Per Hall

**Affiliations:** 1 Human Genetics, Genome Institute of Singapore, 60 Biopolis Street, 138672, Singapore, Singapore; 2 Department of Medical Epidemiology and Biostatistics, Karolinska Institutet, Box 281, 171 77, Stockholm, Sweden; 3 Dr. Margarete Fischer-Bosch Institute of Clinical Pharmacology and University of Tuebingen, Auerbachstr. 112, 70376, Stuttgart, Germany

## Abstract

**Introduction:**

Not all breast cancer patients respond to tamoxifen treatment, possibly due to genetic predisposition. As tamoxifen-induced reductions in percent mammographic density (PMD) have been linked to the risk and prognosis of breast cancer, we conducted a candidate gene study to investigate the association between germline *CYP2D6* polymorphisms and PMD change.

**Methods:**

Baseline and follow-up mammograms were retrieved for 278 tamoxifen-treated subjects with *CYP2D6* metabolizer status (extensive (EM), heterozygous extensive/intermediate (hetEM/IM) or poor metabolizer (PM)). Logistic regression analyses were conducted comparing subjects who experienced >10% reduction in PMD to those who experienced ≤10% reduction or increase.

**Results:**

After multivariate adjustment, PMD change was found to be significantly associated with the degree of *CYP2D6* enzyme functionality (Ptrend = 0.021). Compared with EM, hetEM/IM and PM were 72% (95% confidence interval (CI): 0.10 to 0.79) and 71% (0.03 to 2.62) less likely to experience a >10% reduction, respectively.

**Conclusions:**

Tamoxifen-induced change in PMD appears to have a genetic component.

## Introduction

Tamoxifen reduces both the risk and recurrence of breast cancer [1,2]. It has recently been shown that a decrease in mammographic density predicts response to tamoxifen [3-5]. There may be genetic reasons as to why some women experience a decrease in mammographic density and a dramatic influence on risk and prognosis of breast cancer. While tamoxifen metabolism is complex, it is known that the Cytochrome P450 2D6 (*CYP2D6*) enzyme is necessary to produce clinically active metabolites - 4-hydroxytamoxifen and endoxifen. It has been suggested that patients with no functional *CYP2D6* alleles should be offered alternatives to tamoxifen [6,7]. We thus hypothesize that only women who are able to metabolize tamoxifen would experience a decrease in density and a potential parallel effect on breast cancer risk and prognosis. In this study, we explored the association between *CYP2D6* metabolizer phenotype and mammographic density change, using breast cancer cases from a population-based breast cancer case–control study conducted in Sweden between 1993 and 1995 [8-10].

## Methods

### Study population

Subjects were a subset of the CAncer Hormone Replacement Epidemiology in Sweden (CAHRES) study [8]. Briefly, the parent study consisted of women born in Sweden who were 50 to 74 years old at first diagnosis of invasive breast cancer in the Swedish Cancer Register. Approval for the study was given by the ethical review boards in the respective regions in which the subjects were based: Gothenburg, Linköping, Lund, Umeå, Uppsala and at the Karolinska Institute in Stockholm. Subjects are protected by the informed consent process, in which they were told what was collected and repeatedly given the option of declining to participate. All subjects were informed in writing about the study and that participation was voluntary. The participants have all given their consent in using their DNA for genetic analyses. The process of selection of breast cancer cases included in the current study is summarized in Table 1.

**Table 1:** Flow of patients through the study (inclusion criteria)

	**n**
Genotyped	710
Overlap with women with mammograms retrieved	570
Nonmissing date of initiation of tamoxifen therapy	562
Duration of treatment ≥6 months	530
Complete set of baseline and follow-up mammograms	309
Postmenopausal at baseline	308
Genotyped from blood	278
Baseline percent mammographic density (PMD) ≥10%	186

### DNA source and genotyping

As described previously in [11], DNA was isolated from 3 ml of whole blood with the Wizard Genomic DNA Purification Kit (Promega, Madison, WI, USA) in accordance with the manufacturer's instructions; while DNA from non-malignant cells in paraffin-embedded tissue was extracted by using a standard phenol/chloroform protocol [11,12]. Our initial study set in this biomarker study consisted of 710 tamoxifen-treated breast cancer cases genotyped for *CYP2D6*. DNA from tumor tissue or blood was assayed for polymorphisms associated with reduced (*10, *41) or absent (*3, *4, *5) enzyme activity, as described previously [7]. The *CYP2D6* polymorphisms refer to the CYP Allele Nomenclature Committee (http://www.cypalleles.ki.se) (eTable). Women were classified as having an extensive, heterozygous extensive/intermediate or poor *CYP2D6* metabolism [7].

### Mammogram collection and assessment of mammographic density

Collection of mammograms for the parent study was performed retrospectively. Using national registration numbers [13] that are assigned to all subjects living in Sweden, the current addresses from 1975 to 1995 were obtained for all participants in the parent study through a nationwide population registry. Mammograms were then retrieved from radiology departments conducting screening mammography for those addresses. Mammograms were digitized by the Array 2905HD Laser Film Digitizer, with density resolution set at 12-bit, spatial resolution at 5.0 μm and optical density 0 to 4.7.

Mammographic breast density at baseline (≤1 year prior to initiation of tamoxifen) and 6 to 36 months after treatment was assessed by a fully automated thresholding method [14] and expressed as a percentage of the total breast area (percent mammographic density, PMD). The observed correlation between PMD measured by the current gold standard, a computer-assisted semi-automatic thresholding method named Cumulus, and the automated thresholding method used here was (*r* = 0.88; 95% CI, 0.87 to 0.89) in an external test set [14]. For women with multiple mammograms available within the study period, the image taken closest to the second year after treatment start was selected for follow-up (median time from initiation = 1.96, interquartile range = 0.50).

### Statistical analysis

Our primary endpoint was change in PMD between follow-up and baseline. As change in PMD and absolute mammographic dense area were highly correlated (Spearman’s rho correlation coefficient = 0.89) in our data, we limited our association analysis *CYP2D6* metabolizer status and mammographic density to PMD change. To identify other covariates (confounders) for PMD change, we explored the role of baseline PMD, age at diagnosis (years), body mass index (BMI) (kg/m^2^) at baseline, time difference between baseline and follow-up mammograms (years), history of ever hormone replacement therapy and estrogen receptor (ER) (negative, positive or missing) status using chi-squared and Kruskal-Wallis tests. As there might be mammographic changes caused by other cancer treatments, we also assessed chemotherapy [15] and ever radiotherapy [16,17] as potential confounders. Variables which were found to be significantly associated with PMD change were retained in the final logistic regression model and adjusted for. Linear regression models were initially applied to examine PMD change as a continuous variable. The Wald test was used to determine the statistical significance of an overall linear trend for the association between *CYP2D6* metabolizer status, treated as a semi-continuous variable, and binary PMD change (*P* trend). For univariate analyses involving PMD change (continuous variable), baseline and follow-up PMD, the nonparametric Cuzick’s test for trend was also used (Pnptrend). The goodness-of-fit and proportion of total variance explained by linear regression models with more than one predictor variable were assessed using adjusted R^2^ statistics. Odds ratios (ORs) and 95% confidence intervals (CIs) (lower bound of a 95% confidence interval (L95), upper bound of a 95% confidence interval (U95)) were estimated by applying a logistic regression model, using the group with 10% reduction as a reference. Goodness-of-fit of logistic regression models was tested using the Hosmer-Lemeshow test. A *P* value of ≤0.05 was considered statistically significant. All statistical tests were two-sided. Statistical analyses were performed using R (version 2.15.1) [18].

The dataset consisted of 308 subjects who were postmenopausal at baseline and who underwent treatment for ≥6 months. Genetic variants in *CYP2D6* assayed from tumor-derived DNA are at risk of misclassification due to loss of heterozygosity [19,20], hence we restricted our analysis to 278 patients with genotypes derived from blood.

## Results and discussion

Table 2 shows that PMD change as a binary variable was significantly associated with age (*P* = 0.05) and BMI (*P* = 0.02) at baseline, and strongly associated with baseline PMD (*P* <0.001), results which are corroborated by previous findings [21,22]. Both age and BMI at baseline were also found to be highly significantly associated with baseline PMD (*P* <0.001).

**Table 2:** Description of selected characteristics in subjects

	**PMD reduction ≤10%**	**PMD reduction >10%**	***P***
	**n = 247**	**n = 31**	
*Baseline characteristics*			
Age (years)	63.8 ± 6.2	61.5 ± 5.9	0.05
BMI (kg/m^2^)	25.4 ± 3.7	24.1 ± 4	0.02
HRT ever			0.10
No	106 (42.9)	8 (25.8)	
Yes	141 (57.1)	23 (74.2)	
Baseline PMD	14.7 ± 9.5	32 ± 9.1	<0.001
Time between mammograms (years)	1.9 ± 0.6	2.0 ± 0.5	0.30
*Tumor characteristics*			
ER status			0.15
Positive	167 (67.6)	26 (83.9)	
Negative	29 (11.7)	1 (3.2)	
Missing	51 (20.6)	4 (12.9)	
*Treatment*			
Chemotherapy			0.48
No	236 (95.5)	31 (100)	
Yes	11 (4.5)	0 (0)	
Radiotherapy			0.60
No	159 (64.4)	22 (71)	
Yes	88 (35.6)	9 (29)	
Tamoxifen (mg)			
20	137 (55.5)	13 (41.9)	0.47
40	50 (20.2)	9 (29.0)	
20 and 40	57 (23.1)	9 (29.0)	
*Others	3 (1.2)	0 (0)	

Description of selected characteristics in subjects with less than or greater than a 10% percent mammographic density (PMD) reduction, with *P* values for association. Categorical variables (frequencies (%)) were assessed for association using chi-squared tests, while continuous variables (means ± standard deviation (SD)) were assessed using Kruskal-Wallis tests. *One woman was prescribed an unknown dose of tamoxifen. Two women were prescribed tamoxifen citrate and two were prescribed toremifene, a chlorinated derivative of tamoxifen. HRT, hormone replacement therapy; BMI, body mass index; ER, estrogen receptor.

Results of linear regression analyses treating PMD change as a continuous variable revealed a nonsignificant trend of less PMD decline with reduced *CYP2D6* functionality (*P* = 0.11, Table 3). The variance of PMD change as a quantitative trait explained by *CYP2D6* metabolizer status was modest (approximately 1.0%), although in keeping with most genetic studies of traits assaying common genetic variants of low penetrance [23]. *CYP2D6* metabolizer status was not associated with either baseline or follow-up mammogram alone (Table 3), which agrees with previous findings that PMD does not predict survival when considered at single time points [5,24].

**Table 3:** Results of linear regression analyses

		**Univariate**					**Adjusted**			
	**n**	**Beta**	**SE**	**Pwald**	**Ptrend**	**Pnptrend**	**Beta**	**SE**	**Pwald**	**Ptrend**
PMD change (continuous)*										
EM	122	0.00	Reference		0.053	0.074	0.00	Reference		0.11
hetEM/IM	136	1.47	0.78	0.06			0.87	0.67	0.19	
PM	20	1.89	1.51	0.21			1.61	1.29	0.21	
Adjusted R^2^					**0.010**					0.281
*Baseline PMD†*										
EM	122	0.00	Reference		0.389	0.301	0.00	Reference		0.771
hetEM/IM	136	-0.24	0.17	0.16			-0.17	0.15	0.29	
PM	20	0.00	0.33	1.00			0.11	0.30	0.72	
Adjusted R^2^					0.001					0.162
*Follow-up PMD†*										
EM	122	0.00	Reference		0.831	0.999	0.00	Reference		0.413
hetEM/IM	136	-0.07	0.15	0.64			-0.02	0.14	0.90	
PM	20	0.23	0.30	0.44			0.31	0.28	0.27	
Adjusted R^2^					-0.003					0.100

Results of linear regression analyses treating the absolute difference between follow-up and baseline values in percent mammographic density (PMD) as a continuous variable, and PMD at baseline and follow-up separately. PMD at baseline or follow-up was square-root transformed. *Adjusted for baseline percent mammographic density (PMD), age at baseline (years), and body mass index at baseline (kg/m^2^). †Adjusted for all of the above except for baseline PMD. SE, standard error; Pwald, *P* value from Wald tests; Ptrend, *P* value from Wald tests treating genotypes (0, 1, 2) as a continuous variable; Pnptrend, *P* value from Cuzick’s test for trend; EM, extensive metabolizer; hetEM/IM, heterozygous extensive/intermediate; PM, poor metabolizer; adjusted R^2^, adjusted R^2^ statistic.

**Figure 1:**
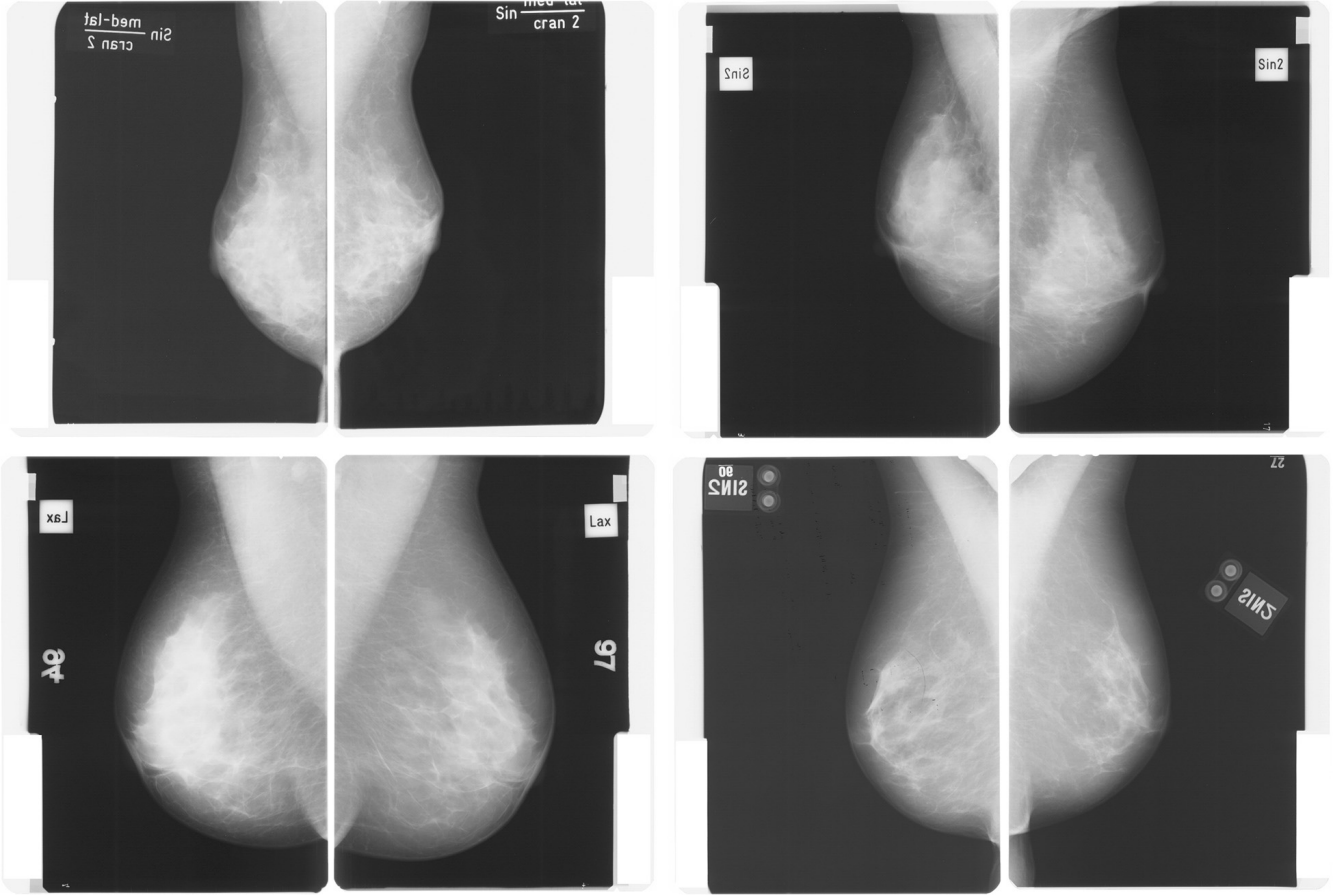
Examples of approximately 10% percent mammographic density reduction from baseline (left) to follow-up (right) mammograms of the same breast.

As the use of categorical cutoff points allows for an easy application and interpretation, we *a priori* specified and dichotomized the difference between PMD at follow-up and baseline into ≤10% reduction and >10% reduction. A PMD change of 10% has been reported to be the minimum difference that could be reproducibly detected by visual assessment [3,4]. Four examples of approximately 10% PMD reduction are shown in Figure 1.

**Figure 2:**
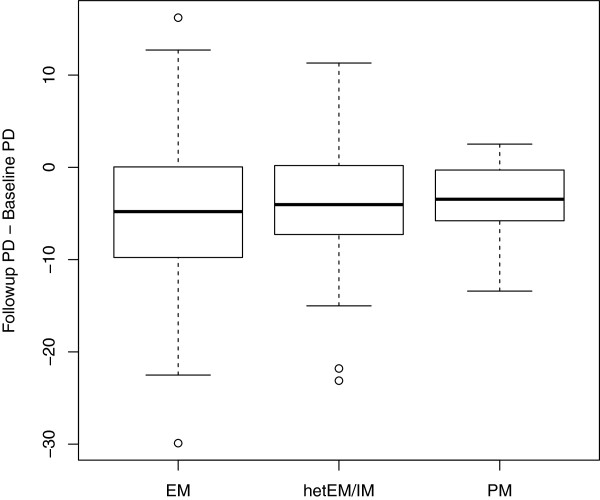
**Boxplot of percent mammographic density (PMD) change by *CYP2D6 *metabolizer status.** Boxplot of percent mammographic density (PMD) change by *CYP2D6* metabolizer status for subjects genotyped from whole blood and with baseline PMD ≥10% (n = 186). The maximum length of each whisker is 1.5 times the interquartile range. EM, extensive metabolizer; hetEM/IM, heterozygous extensive/intermediate; PM, poor metabolizer.

When using this cutoff, our data is consistent with the hypothesis that women with reduced *CYP2D6* function are less likely to experience a decline in PMD. PMD change during tamoxifen therapy was found to be significantly associated with the degree of functionality of *CYP2D6* (Ptrend = 0.020, Table 4). Compared with women who were extensive metabolizers of tamoxifen, intermediate and poor metabolizers were 72% (95% CI: 0.10 to 0.79) and 71% (0.03 to 2.62) less likely to experience a >10% reduction, respectively. As it is difficult for women with low baseline PMD to experience a >10% PMD change, we repeated the analyses for a subgroup of women with baseline PMD ≥10% (n = 186). The significant association between *CYP2D6* metabolizer status and PMD change persisted (Ptrend = 0.021, Table 4). Further adjustment of other potential confounders did not change the estimates appreciably (data not shown). Given the current discussion on genotype-driven tamoxifen dosing [25,26], we further adjusted for drug dosage to account for effective therapeutic metabolite ranges of different *CYP2D6* metabolizer status, but did not observe an appreciable change in the results (data not shown). The boxplot in Figure 2 shows graphically that PMD reduction was weaker with genetic *CYP2D6* deficiency. It has recently been shown that mammographic density at time of breast cancer diagnosis did not influence breast cancer prognosis [5,24], but while mammographic density did not have a bearing on survival when considered singly at snapshot time points, we reported that a pronounced decrease in mammographic density between baseline and follow-up mammograms following tamoxifen treatment was found to significantly reduced the risk of dying from breast cancer in a previous study [5]. An independent study in an Asian population also showed that mammographic density change during short-term use of adjuvant endocrine therapy was also found to be a significant predictor of long-term recurrence in women with ER-positive breast cancer [3]. It is conceivable that the association between PMD change and breast cancer survival in patients treated with tamoxifen could be explained by differences in compliance or in this case, an inherited response to treatment (for example due to genetic variation in the *CYP2D6* gene).

**Table 4:** **Results of tests of association between *CYP2D6 *** **metabolizer variants and change in percent mammographic density**

	**PMD reduction**		**Univariate**				**Adjusted**			
	**≤10%**	**>10%**	**OR**	**L95**	**U95**	**Ptrend**	**OR**	**L95**	**U95**	**Ptrend**
+ Genotyped from whole blood										
EM	101	21	1.00	Reference		0.009	1.00	Reference		0.020
hetEM/IM	127	9	0.34	0.15	0.78		0.28	0.10	0.79	
PM	19	1	0.25	0.03	2.00		0.29	0.03	2.62	
n = 278										
GOF										0.928
+ Genotyped from whole blood										
+ Baseline PMD ≥10%										
EM	63	21	1.00	Reference		0.010	1.00	Reference		0.021
hetEM/IM	79	9	0.34	0.15	0.80		0.29	0.11	0.81	
PM	13	1	0.23	0.03	1.87		0.29	0.03	2.57	
n = 186										
GOF										0.609

Results of tests of association between *CYP2D6* metabolizer variants and change in percent mammographic density (PMD) in subjects treated with tamoxifen for at least six months. Mammograms were taken ≤1 year prior to initiation of tamoxifen, up to three years after treatment. The mammogram taken closest to the second year after tamoxifen treatment was selected for follow-up. Odds ratios (ODs) and 95% confidence intervals (CIs) (lower bound of a 95% confidence interval (L95), upper bound of a 95% confidence interval (U95)) were estimated by applying a logistic regression model, using the group with 10% reduction as a reference. Analyses were adjusted for baseline PMD, age at diagnosis (years), and body mass index (kg/m^2^) at baseline. The Wald test was used to determine the statistical significance of an overall linear trend for the association between *CYP2D6* metabolizer status, treated as a semi-continuous variable, and PMD change (Ptrend). All statistical tests were two-sided. Statistical analyses were performed using R (version 2.15.1) [18]. EM, extensive metabolizer; hetEM/IM, heterozygous extensive/intermediate; PM, poor metabolizer; GOF, *P* value for Hosmer-Lemeshow goodness-of-fit test for logistic regression.

Nonetheless, two large trials have previously reported a null association between *CYP2D6* genotype and breast cancer outcome [27,28], which appears to contradict our findings. The validity of the two studies, however, has been questioned due to the quality of genotyping on the basis of massive departures from Hardy-Weinberg equilibrium and the use of tumor tissue [29]. In this study, we have addressed the concerns and restricted our analysis to germline DNA extracted from blood. The individual genotype counts of *CYP2D6* metabolizer status were also presented in the results.

To the best of our knowledge, this is the first time that *CYP2D6* has been shown to influence PMD change. A major strength is the comprehensive assembly of various datasets, which helped to give rise to a new mechanistic insight on a putative relationship between active metabolites, as predicted by CY2D6 metabolizer status, and local morphological changes. Using a fully automatic thresholding method to measure PMD on serial mammograms adds to the strength of our study as intra- and interreader variability was substantially reduced. The method produces one reproducible reading per mammogram; unlike other user-dependent methods, in which readings can vary when a mammogram is reread by the same user, or read by another user. However, due to a limitation in size of the study, validation of our findings in an independent dataset is of critical importance. A lack of adequate data prevents the ruling out of the impact of *CYP2D6* inhibitors on the results.

## Conclusions

In conclusion, our exploratory study has yielded preliminary evidence to show that there is a significant association between *CYP2D6* metabolizer status and PMD change, suggesting that the capacity for PMD change may be inherited. Although the sample size is limited, our finding may still be clinically important and warrant further consideration. The question of whether *CYP2D6* could be used as a risk or prognostic marker for mammographic density changes in response to tamoxifen treatment will require larger prospective studies.

## Abbreviations

BMI: Body mass index; CAHRES: Cancer hormone replacement epidemiology in Sweden; CI: Confidence interval; CYP2D6: cytochrome P450 2D6; EM: Extensive metabolizer; ER: Estrogen receptor; hetEM/IM: Extensive/intermediate metabolizer; L95: Lower bound of a 95% confidence interval; OR: Odds ratio; PM: Poor metabolizer; PMD: Percent mammographic density; SD: Standard deviation; U95: Upper bound of a 95% confidence interval.

## Competing interests

The authors declare that they have no competing interests.

## Authors’ contributions

PH conceived of the study. PH, KC, KH, YL and JL participated in the design of the study and performed the statistical analysis. PH, KC, KH, JL, WS, HB and PS participated in its design and coordination and helped to draft the manuscript. All authors read and approved the final manuscript.
